# Central control of body temperature

**DOI:** 10.12688/f1000research.7958.1

**Published:** 2016-05-12

**Authors:** Shaun F. Morrison

**Affiliations:** 1 Department of Neurological Surgery, Oregon Health & Science University, Portland, OR, USA

**Keywords:** Brown adipose tissue, shiver, cutaneous vasoconstriction, thermogenesis, fever, sympathetic nerve activity, preoptic hypothalamus, rostral raphe pallidus, dorsomedial hypothalamus, therapeutic hypothermia, obesity

## Abstract

Central neural circuits orchestrate the behavioral and autonomic repertoire that maintains body temperature during environmental temperature challenges and alters body temperature during the inflammatory response and behavioral states and in response to declining energy homeostasis. This review summarizes the central nervous system circuit mechanisms controlling the principal thermoeffectors for body temperature regulation: cutaneous vasoconstriction regulating heat loss and shivering and brown adipose tissue for thermogenesis. The activation of these thermoeffectors is regulated by parallel but distinct efferent pathways within the central nervous system that share a common peripheral thermal sensory input. The model for the neural circuit mechanism underlying central thermoregulatory control provides a useful platform for further understanding of the functional organization of central thermoregulation, for elucidating the hypothalamic circuitry and neurotransmitters involved in body temperature regulation, and for the discovery of novel therapeutic approaches to modulating body temperature and energy homeostasis.

Body temperature (T _core_) is a critical homeostatic parameter influencing cellular function and organismal survival. Life-threatening protein denaturation looms as T _core_ increases, and reductions in membrane fluidity, ion fluxes, and enzyme performance accompany significant reductions in T _core_. The fundamental central neural circuits for thermoregulation orchestrate behavioral and autonomic repertoires that maintain T _core_ during thermal challenges, sensed by thermal receptors, that arise from either the ambient or the internal (e.g., during exercise) environment. A variety of other neural circuits and neurochemical modulators impinge on the fundamental thermoregulatory pathways to produce the alterations in T _core_ that occur with circadian and ultradian periodicities ^1, 2^ and those that accompany many behavioral states, such as fever during sickness, increased T _core_ during psychological stress and at ovulation, and reductions in T _core_ during sleep, sepsis, or exposure to metabolic distress (e.g., hypoxia and starvation). Recent research in the central nervous system (CNS) control of T _core_ focuses principally on: (a) elaborating the CNS pathways and neurotransmitter systems involved in the fundamental central thermoregulatory network; (b) the modulation of activity within the fundamental central thermoregulatory network by non-thermal factors and behavioral states; and (c) pharmacological manipulation of the CNS thermoregulatory network for a variety of therapeutic goals (e.g., reducing T _core_ to produce therapeutic hypothermia in stroke victims).

In its simplest form, the fundamental thermoregulatory network can be modeled as a reflex ^3, 4^ in which a central integrative circuit alters the activity of thermoeffector mechanisms in response to an input from the combination of peripheral (i.e., skin) and central (i.e., visceral and brain) thermoreceptors that provides a consolidated assessment of T _core_ and, importantly, of imminent threats to T _core_. The primary thermoeffector mechanisms recruited for both cold defense and centrally driven hyperthermias (e.g., fever) include: (a) thermoregulatory behaviors to reduce the loss of heat produced during basal metabolism; (b) cutaneous vasoconstriction (CVC) to conserve heat in the body core and limit heat loss to the environment; and (c) heat production (thermogenesis). The principal sources of metabolic heat production, beyond those contributing to basal metabolic rate (e.g., pumping ions across membranes), are brown adipose tissue (BAT), whose sympathetic neural input fuels mitochondria that shunt proton fluxes into heat production, and shivering behavior in skeletal muscle, dependent on the inefficiency of ATP utilization to generate heat. Effector mechanisms for heat defense include: (a) thermoregulatory behavior to increase heat loss; (b) cutaneous vasodilation, which in humans includes a sympathetic vasodilator outflow ^5, 6^, combined with increased cardiac output ^7^ and visceral vasoconstriction ^8– 10^ to facilitate core heat loss from the body surface; and (c) evaporative cooling (e.g., sweating).

Apart from important “first responders”, the CNS pathways controlling and mediating thermoregulatory behaviors remain incompletely defined ^11– 15^. Along these same lines, most research subjects are furry mammals that are strongly dependent on non-sweating mechanisms for evaporative cooling. Although this has limited the available detail on the CNS pathways regulating human sweating ^6, 16, 17^, considerable insight has been gained on the CNS regulation of other mechanisms of evaporative cooling, such as panting ^18– 20^. Additionally, since most of the basic neuroscience of CNS thermoregulatory pathways has been derived from experiments in rodents, including the exclusively *in vitro* studies of neurons with intrinsic thermosensitivity, the translation of the conclusions, including the circuit model in Figure 1, to humans must be cautiously undertaken (e.g., 21). Finally, although considerable progress has been achieved in the relatively young field of the neuroscience of thermoregulation, the synthesis ( Figure 1) of our understanding of this multifaceted neural network controlling multiple thermoeffectors represents a working model, with the expectation of revisions and added detail.

**Figure 1.**
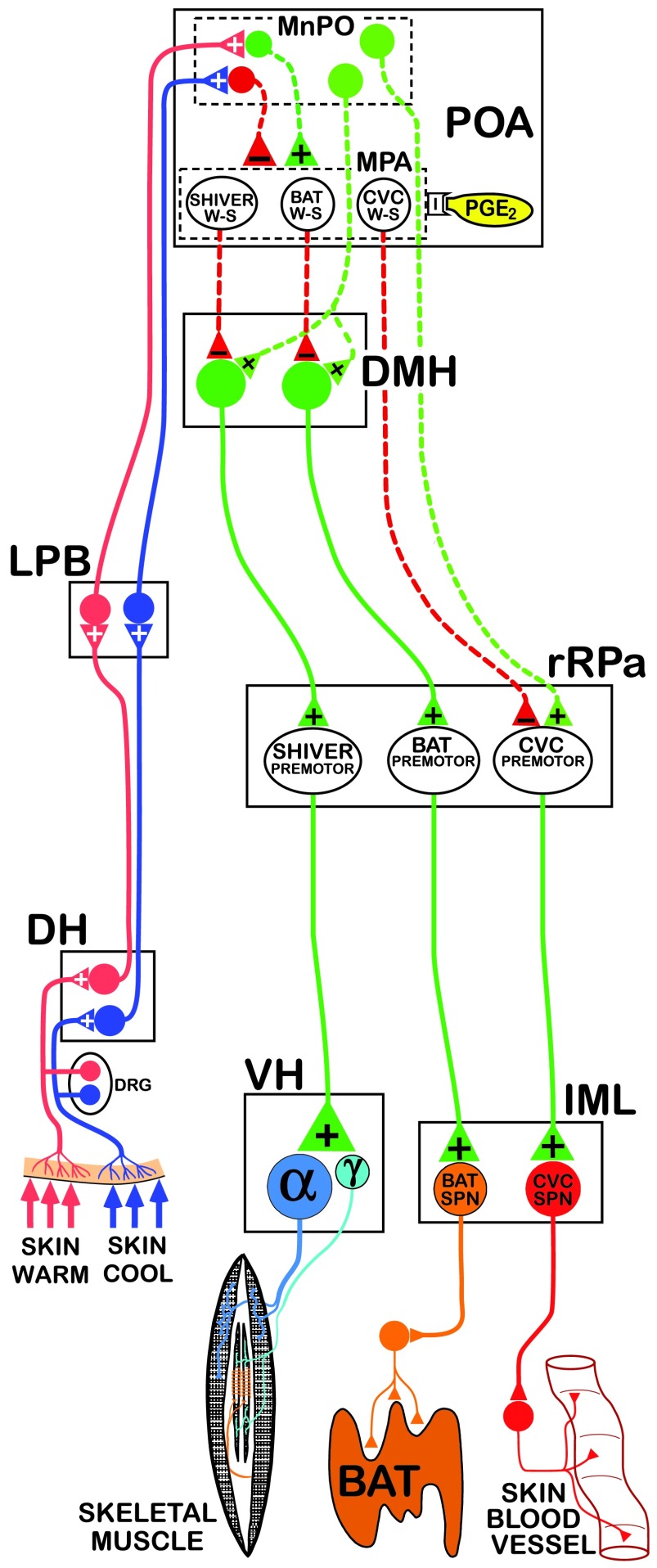
Functional neuroanatomical model for the fundamental pathways providing the thermoregulatory control and pyrogenic activation of cutaneous vasoconstriction (CVC) and brown adipose tissue (BAT) and shivering thermogenesis. Cool and warm cutaneous thermoreceptors transmit signals to respective primary sensory neurons in the dorsal root ganglia (DRG) which relay this information to second-order thermal sensory neurons in the dorsal horn (DH). Cool sensory DH neurons glutamatergically activate third-order sensory neurons in the external lateral subnucleus of the lateral parabrachial nucleus (LPB), while warm sensory DH neurons project to third-order sensory neurons in the dorsal subnucleus of the LPB. Thermosensory signals driving thermoregulatory responses are transmitted from the LPB to the preoptic area (POA), which contains the microcircuitry through which cutaneous and core thermal signals are integrated to regulate the balance of POA outputs that are excitatory (dashed green) and inhibitory (dashed red) to thermogenesis-promoting neurons in the dorsomedial hypothalamus (DMH) and to CVC sympathetic premotor neurons in the rostral raphe pallidus (rRPa). Within the POA, GABAergic interneurons (red) in the median preoptic (MnPO) subnucleus are postulated to receive a glutamatergic input from skin cooling-activated neurons in LPB and inhibit each of the distinct populations of warm-sensitive (W-S) neurons in the medial preoptic area (MPA) that control CVC, BAT, and shivering. In contrast, glutamatergic interneurons (dark green) in the MnPO are postulated to be excited by glutamatergic inputs from skin warming-activated neurons in LPB and, in turn, excite the populations of W-S neurons in MPA. Prostaglandin E _2_ (PGE _2_) binds to EP3 receptors, which are postulated to inhibit the activity of each of the classes of W-S neurons in the POA. Preoptic W-S neurons may provide inhibitory control of CVC by inhibiting CVC sympathetic premotor neurons in the rostral ventromedial medulla, including the rRPa, that project to CVC sympathetic preganglionic neurons (SPNs) in the intermediolateral nucleus (IML). Preoptic W-S neurons may provide inhibitory thermoregulatory control of BAT and shivering thermogenesis by inhibiting BAT sympathoexcitatory neurons and shivering-promoting neurons, respectively, in the DMH, which, when disinhibited during skin and core cooling, provide respective excitatory drives to BAT sympathetic premotor neurons and to skeletal muscle shivering premotor neurons in the rRPa. These, in turn, project, respectively, to BAT SPNs in the IML and to alpha (α) and gamma (γ) motoneurons in the ventral horn (VH) of the spinal cord.

The CNS thermoregulatory control of the sympathetic outflows mediating CVC and BAT thermogenesis and of the somatic motoneurons producing shivering is effected through parallel but distinct, effector-specific, integrative/efferent circuits ( Figure 1, and reviewed in 22– 25) that share common peripheral thermal sensory inputs. The hypothalamus contains the primary integrative and rostral efferent components of these circuits. Although many details of the preoptic area (POA) microcircuitry for thermoregulation remain to be elucidated, neurons in the POA are postulated to integrate ascending peripheral thermosensory signals with local thermosensitivity to regulate the output of BAT and shivering thermogenesis-promoting neurons in the dorsomedial hypothalamus (DMH) ^26, 27^ and of CVC-promoting neurons in the median preoptic nucleus (MnPO) ^28, 29^.

The POA regulation of DMH thermogenesis-promoting neurons represents the balance between a GABAergic inhibition ^30, 31^ and a glutamatergic excitation ^32^; the latter inputs, potentially arising from neurons in the MnPO that project to the DMH, are synaptically connected to BAT ^33^ and express the leptin receptor ^34^. These glutamatergic inputs to DMH ^32^ could provide the excitation required to drive the BAT sympathoexcitatory neurons and the shivering-promoting neurons in DMH when their POA inhibitory input is reduced during skin cooling or fever ^35^. Although intrinsically warm-sensitive (W-S) ( Figure 1), POA neurons ^36– 38^, generally located in the medial preoptic area (MPA) ^39^, are postulated to play a key role in central thermoregulation by providing a prominent core temperature-modulated, GABAergic ^38^ regulation of thermogenesis-promoting neurons in DMH ( Figure 1), the considerable direct functional evidence required to establish this attractive hypothesis has yet to be obtained. Different thermal sensitivities or neurochemical modulation among populations of temperature-sensitive POA neurons may underlie the differential responsiveness of different effectors to changes in cutaneous versus brain temperatures ^40^ as well as the significant alterations in thermoeffector activation during different sleep phases ^41^. Through their responses to immune signaling molecules, neurons in the POA are also the primary site for the organization and maintenance of the febrile response to inflammation and infection, which includes the stimulation of CVC, and shivering (“chills”) and BAT thermogenesis mediated by the action of prostaglandin E _2_ (PGE _2_) on its EP3 receptors ^42– 45^. Similarly, the fundamental thermoregulatory network mediates stress-induced hyperthermia ^46– 48^. Unraveling the complexity of the thermoregulatory circuitry in the hypothalamus ^20, 28, 29, 49– 52^, including the phenotypic characterization of the projection neurons ^34^ and their synaptic interactions that mediate the circadian ^13^ and many behavioral ^53, 54^ modulations in T _core_, continues to pose significant research challenges for understanding the “heart” of the CNS thermoregulatory network. The downstream targets of the hypothalamic projection neurons for thermoregulation are the sympathetic and somatic premotor neurons in the rostral ventromedial medulla, centered on the rostral raphe pallidus (rRPa). Midbrain (e.g., periaqueductal gray ^55– 57^ and retrorubral field ^58^) and medullary (e.g., ventrolateral medulla and nucleus of the solitary tract (NTS) ^59, 60^) pathways to these premotor neurons exert important modulatory influences on the thermoregulatory activation of thermoeffector organs (reviewed in 22, 24). These premotor neurons, in turn, excite CVC and BAT sympathetic preganglionic neurons and α-motoneurons and γ-motoneurons ^61, 62^ in the spinal cord.

Cold and warm thermoreceptors in the skin and viscera provide the extracranial thermal signals relating to skin temperature and T _core_. These are integrated with the brain temperature information potentially derived from the discharge of W-S, GABAergic preoptic neurons of the central thermoregulatory network to regulate thermoeffector activities. The membranes of thermoreceptor afferent neurons contain transient receptor potential (TRP) cation channels whose temperature-dependent conductances transduce skin temperature into primary thermoreceptor afferent neuronal activity. The TRPM8 channel, activated by menthol and cooling, is the strongest candidate for the cutaneous cold receptor (reviewed in 63). The identity of the peripheral warm receptor remains to be established, but the warm-sensing mechanism of preoptic neurons, though still debated ^64– 66^, is unlikely to involve a transient receptor potential (TRP) channel ^67^. Primary thermoreceptor dorsal root ganglion neurons synapse on thermoreceptive-specific, lamina I spinal (or trigeminal) dorsal horn cells ^68^, and these, in turn, collateralize to innervate the thalamus, providing the neural substrate for cutaneous thermosensory perception and localization ^68, 69^, and the pontine lateral parabrachial nucleus (LPB) ^70, 71^, which is responsible for triggering involuntary (e.g., autonomic and shivering) thermoregulatory responses. Spinal lamina I skin cooling-responsive neurons provide a glutamatergic excitation to neurons in the external lateral subdivision of the lateral parabrachial nucleus (LPBel), which, in turn, project principally to the MnPO of the POA ^72, 73^, while a parallel, spinoparabrachial glutamatergic pathway excites POA-projecting neurons in the dorsal subnucleus of the LPB (LPBd) ^72, 74^ in response to skin warming ^74^. Thus, activations of POA-projecting LPBd and LPBel neurons, driven respectively by cutaneous, and likely visceral, warm and cold thermoreceptor stimuli, initiate heat defense and cold defense inhibitions and excitations, respectively, in CVC sympathetic outflow and cutaneous blood flow, in BAT sympathetic outflow and BAT thermogenesis, and in shivering EMGs and shivering thermogenesis to maintain a homeostatic T _core_.

Synaptic integration sites throughout the core thermoregulatory network provide the substrate for a wide variety of non-thermal physiological parameters, disease processes, neuromodulators, and drugs to influence the central regulation of T _core_ (reviewed in 25). For example, the high metabolic rate of BAT and shivering skeletal muscles during thermogenesis cannot be sustained without a dependable supply of metabolic fuels, particularly oxygen ^75^, lipolytic by-products ^34^, and glucose ^76^. Thus, the CNS networks driving cold-defensive and behavioral BAT activation or shivering are strongly inhibited by signals reflecting a reduction in the short- and long-term availability of these fuel molecules essential for BAT and skeletal muscle metabolism. Similarly, as hypovolemia progresses during dehydration, the ensuing hyperosmolarity reduces the thermoregulatory drive for sweating ^20, 77, 78^, which serves to prevent cardiovascular collapse ^79^. Some viscerosensory afferents with axons in the vagus nerve and synapsing on second-order neurons in the NTS can also influence BAT activity ^25, 80^ and shivering responses and thus are expected to influence the regulation of T _core_. For instance, vagal afferents convey the “metabolic” signals that produce the inhibition of BAT activity induced by upregulation of hepatic glucokinase ^81^ and the BAT activation following either intragastric delivery of the TRP agonist, capsiate ^82^, or the presence of lipids in the duodenum ^83^. Another influence on T _core_ includes a prominent hypothermia during motion sickness and nausea ^84^, for which the pathways relating vestibular stimulation to inhibition of CVC and thermogenesis remain to be elaborated.

Providing an additional layer of complexity in the CNS regulation of T _core_ is the presence of the thermally responsive TRPV1 channel in the membranes of a variety of classically non-thermal, unmyelinated afferents ^85– 88^ that may have access to central thermoregulatory circuits, including via NTS neurons that can inhibit thermogenesis ^59^. The finding that the TRPV1 responsiveness to local brain temperature alters spontaneous glutamate release from the terminals of unmyelinated vagal afferents ^88, 89^ provides a potential substrate for T _core_ to modulate the activity of second-order sensory neurons in NTS that modulate thermoeffector activation. Importantly, TRPV1 channels are also activated by non-thermal factors, including low (or high) pH, inorganic cations, or endovanilloids, thereby providing a basis for such factors to influence thermoeffector activation and thus T _core_. For instance, TRPV1 channels, potentially on the terminals of afferents in the peritoneum, are stimulated by an endogenous ligand to tonically inhibit BAT thermogenesis, which results in a hyperthermic response to TRPV1 antagonist administration ^85, 90^. Although these afferents were not directly tested for their thermal responsiveness, the hyperthermic response to TRPV1 antagonism was not altered by changes in T _core_. The relative influences and the interactions of thermal and non-thermal stimuli on the conductance of the relevant TRPV1 channels could play a role in determining their effect on the level of thermoeffector activation to thermoreceptor stimuli.

Interest in pharmacological modulation of the central thermoregulatory network has focused: (a) on reducing CVC and thermogenesis to lower T _core_; and (b) on augmenting thermogenesis to elevate energy expenditure with the goal of weight loss through consumption of the high-energy lipid stores in white adipose tissue. Novel approaches to reducing T _core_ would have immediate benefits in treating intractable fevers that are unresponsive to cyclooxygenase inhibitors. Therapeutic hypothermia can have beneficial effects on survival and on reducing brain and tissue damage in ischemic insults such as cardiac arrest, stroke, and neonatal encephalopathy ^91– 94^. Extended space travel (e.g., Mars One) also may require pharmacological induction of a hypothermic, hibernation-like state to reduce energy consumption and psychological stress.

Under basal metabolic and movement conditions, changes in T _core_ must arise from changes in the level of activation of thermoeffector tissues. Although modulating neuronal discharge at any site within the central thermoregulatory network would be expected to alter T _core_, centrally generated hyperthermias, such as fever, that arise from altered activity in hypothalamic thermoregulatory neurons could be most effectively reduced by manipulating thermoeffector efferent pathways ^95, 96^. Indeed, directly inhibiting the discharge of neurons in the rRPa area, including the functionally significant premotor neurons that control the principal thermoeffectors, produced a fall in brain temperature of approximately 14°C in an ambient environment of 15°C ^97^, and stimulation of α2 adrenergic receptors in the rRPa could completely block or prevent a lipopolysaccharide-evoked fever ^95^. Similarly, central administration of an adenosine A1 receptor (A1AR) agonist reduced rat T _core_ by 10°C in an ambient temperature of 15°C ^60^ and mouse T _core_ by 5°C in an ambient temperature of 4°C ^98^. Of particular interest, in rats, this hypothermia, which was produced by a blockade of the cold-evoked activation of BAT and shivering thermogenesis, was long-lasting and paralleled by marked reductions in heart and respiratory rate, in EEG, and in behavior ^60^—all reminiscent of those occurring in hibernation and torpor, which require central stimulation of the A1AR ^99– 101^. The discovery of a role for TRP channels in thermal sensation and in thermoeffector activation has stimulated research into the pharmacological modulation of TRP channels in the central thermoregulatory network to abrogate the normal cold defense mechanisms and allow T _core_ to fall in a cool ambient environment. By reducing the activation of cooling-responsive skin thermoreceptors, a TRPM8 antagonist reduced rat T _core_ by approximately 1°C when the rats were in an ambient temperature of 19°C ^63^. Stimulating TRPV1 reduced mouse T _core_ by approximately 12°C when mice were exposed to a 10°C ambient temperature ^102^, and this effect was potentiated by the addition of a TRPM8 antagonist ^103^. The location of the relevant, hypothermic TRPV1 channels remains unknown.

Not only is BAT a thermogenic thermoeffector, including in adult humans ^104– 107^, but through its consumption of lipid and glucose energy stores and oxygen, thermogenic metabolism in BAT is a neurally regulated contributor to energy homeostasis. Thus, particularly in the face of an elevated consumption of energy-rich food (e.g., a high-fat diet), a chronic reduction in cooling-evoked BAT thermogenesis would contribute to the augmented adipose energy stores that characterize obesity. Indeed, mice without BAT exhibit a propensity for obesity and diabetes ^108, 109^; conversely, overexpression of uncoupling protein-1 (UCP-1), principally responsible for thermogenesis in BAT, mitigates obesity induced by a high-fat diet ^110^. Several anti-obesity therapies currently being explored are based on increased activation of BAT thermogenesis, through either activation of the central thermoregulatory network to increase the sympathetic outflow to BAT ^111^ or an alteration in the cellular biochemical pathways in brown adipocytes or a hyperplasia of BAT to augment thermogenesis. The consistent findings that obese humans have significantly reduced cooling-activated BAT ^104– 106, 112^, and that the basal (i.e., principally cooling-evoked) sympathetic activation of BAT is reduced in rats fed a high-fat diet ^113, 114^, and that a vagal afferent input to the NTS mediates the reduced cooling-evoked BAT activity in rats fed a high-fat diet ^115^ not only support a role for reduced BAT activity in the excess adipose accumulation of obesity but also highlight the significance of non-thermal inputs to the central thermoregulatory network ^25, 81, 83^ in influencing even the most basic thermoregulatory responses.

Considerable progress has been achieved in revealing the functional organization of the dedicated thermoregulatory network within the CNS that provides the fundamental neural control of the thermoregulatory effectors: thermoregulatory behavior, CVC, and BAT and shivering thermogenesis, although many of the details of the neurophysiology and neuroanatomy of the central thermoregulatory network remain active areas of investigation. The changes in T _core_ that accompany a wide range of behaviors and in response to many hormones and drugs arise through altered non-thermal inputs to, or neurochemical modulation of, the neural activity within the fundamental thermoregulatory network. The latter, as well as thermoreceptor-based strategies, are being researched as therapeutic approaches in which the central thermoregulatory networks are recruited to alter T _core_ and metabolism.
